# Using the Three Delays Model to Examine Civil Registration Barriers in Indonesia

**DOI:** 10.1371/journal.pone.0168405

**Published:** 2016-12-19

**Authors:** Cyril Bennouna, Brooke Feldman, Rahmadi Usman, Rama Adiputra, Santi Kusumaningrum, Lindsay Stark

**Affiliations:** 1 Center on Child Protection and Wellbeing, Universitas Indonesia (PUSKAPA), School of Social and Political Sciences, Depok, Indonesia; 2 Program on Forced Migration and Health (PFMH), Mailman School of Public Health, Columbia University, New York, NY, United States of America; TNO, NETHERLANDS

## Abstract

The Three Delays Model has proven a useful framework for examining barriers to seeking obstetric care and preventing maternal and child mortality. This article demonstrates the applicability of the Three Delays Model to the case of civil registration in rural Indonesia and examines ways that efforts to strengthen civil registration services can draw on lessons from maternal and child health programming. Twenty focus group discussions were conducted using a participatory ranking exercise in four Indonesian districts. Focus groups were stratified into four groups: (1) government officials involved in civil registration, (2) civil society organization members that assist communities in civil registration, and (3) female and (4) male community members. Transcripts were analyzed using constant comparative method and thematic analysis, revealing barriers that communities commonly faced in accessing civil registration services. In examining the categories and themes related to these barriers, the research team found a significant overlap with the factors and phases of the Three Delays Model. Participants were delayed from seeking registration services by a range of sociocultural factors and by the perceived inaccessibility and poor quality of services. Once they decided to seek care, long distances to services and poor transportation options delayed their access to registration offices. Finally, a series of bottlenecks in service provision created extended delays once applicants reached registration offices. Ownership of civil registration documents in Indonesia remains exceptionally low, with just over half of children and youth possessing a birth certificate. To strengthen civil registration and health systems more generally, it is important to understand the factors that enable and constrain civil registration, how these factors relate to one another, and how they change over a child’s life.

## Introduction

Around the world, nearly one in three children under the age of five do not have a birth certificate [1]. Researchers at the World Health Organization have referred to the enormous gap in civil registration coverage globally as “the single most critical development failure over the past 30 years” [2]. Countries such as Indonesia, with decentralized authority, large rural population, and remote communities, have struggled to expand access to quality civil registration services. As a result, large domestic inequities in birth certificate ownership persist, depending on factors such as urbanization, income, and education [3, 4]. A 2013 review, for example, found that the proportion of Indonesian children without birth certificates in rural areas was twice that of children in urban areas [4]. Living in the poorest quartile also significantly decreased children’s chances of having a birth certificate, in some provinces by a factor of nine. Another study reported that application costs, distance from services, and the complexity of application procedures were the three most critical barriers to obtaining birth certificates in Indonesia [5].

Addressing these inequities in civil registration is a key component of the Indonesian government’s Medium Term Development Plan, which aims to increase birth certificate coverage to 85 percent of all children under 18 years old by 2019, with a specific focus on vulnerable populations [6]. According to the Indonesian Central Statistics Agency (BPS)’s most recent national estimate, the current coverage for children across the country is 63 percent [7]. Narrowing this coverage gap, and strengthening the country’s civil registration system more generally, will require a more comprehensive understanding of the dynamic factors that enable and constrain birth registration for Indonesia’s vulnerable populations.

In addition to being a development priority, strengthening civil registration is increasingly seen as a core investment in public health as well [2]. Civil registration documents can enable individuals to access basic services, and in turn civil registries can feed into national vital statistics systems, which allow government bodies to plan health programs and monitor progress more effectively. Without comprehensive and timely birth registration, for example, it can be challenging to surveil fertility and fertility-related trends, such as child mortality [8]. Countries with stronger civil registration and vital statistics tend to have better health outcomes, including lower maternal mortality ratios and child mortality risk [9]. In Indonesia, although the health sector has no formal role in birth registration, studies have found children’s birth certificate ownership to be positively associated with being delivered in a health facility or by a skilled health attendant, and use of postnatal care [10].

With the Indonesian government’s renewed commitments to improving civil registration and maternal and child health under its National Medium Term Development Plan and the Sustainable Development Goals (SDGs), locating birth registration within a healthcare framework can help to reinterpret the challenges the country faces and can inform new strategies that strengthen both systems at once [6]. Thaddeus and Maine’s Three Delays Model may provide a useful conceptual framework for understanding the relationships between the barriers to civil registration in Indonesia [11]. The model was originally developed to characterize factors contributing to maternal mortality between the onset of an obstetric complication and its outcome. Similar to the case of birth registration, maternal health studies that preceded the Three Delays Model had identified cost, distance, and quality of care as barriers to seeking obstetric care, but lacked a broader framework to explain the relationships between these barriers, and to contextualize them within the larger system failures that made quality care inaccessible to many community members. In addition to providing a useful heuristic for interpreting the barriers to civil registration, the Three Delays Model also offers an opportunity to locate intersections between Indonesia’s civil registration and vital statistics system and its healthcare system and to identify ways these systems can mutually reinforce one another.

### Guiding Framework: The Three Delays Model

The Three Delays Model is comprised of three phases. Each phase is modified by factors affecting utilization and outcomes, including socioeconomic and culture factors, accessibility, and the quality of services. Phase I centers on the decision to seek care, which can be influenced by societal norms and poor knowledge. Resources such as time and money often limit the ability of those who intend to use social services. Phase II includes identifying and traveling to facilities, and is influenced by the distance and transportation infrastructure between villages and the appropriate facilities. Phase III involves receiving adequate and appropriate care at facilities. Factors in this phase affect both utilization of services and outcomes. Provider competency, availability of commodities, and communication influence this third phase.

The Three Delays Model has been widely used across development settings from Malawi to Haiti to Indonesia to explain high rates of maternal mortality [12–16]. In India, Tanzania, and Uganda, the model was expanded to inform interventions to reduce neonatal and child mortality [17–19]. Evidence has shown the great value of this model in a variety of contexts. Although it has usually been applied to understand factors related to emergency medical services, many similar social, behavioral, and systems factors constrain access to non-emergency services, such as civil registration. This study drew on focus group discussions to explore the applicability of the Three Delays Model to understand shortcomings in Indonesia’s birth registration system. The application of this model to the context of civil registration may contribute to theorizing solutions for Indonesia’s enduring birth registration gap.

## Methods

### Setting

Qualitative data were collected from four districts across North Sumatra (SUMUT) and West Nusa Tenggara (NTB) in June and July 2015. SUMUT, a large, mountainous, and heavily forested area, is the fourth most populous province in Indonesia, with over 13.5 million people [20]. About 10 percent of SUMUT’s population lives below the village-based Indonesian poverty line, slightly better than the national average of 14 percent. Langkat is the northernmost district in the province with a population of just under one million. Asahan is southeast, located on the coast of the Strait of Malacca, with a slightly smaller population of about 670,000. Both districts are predominantly Muslim with a diverse ethnic makeup comprised of Malays, Chinese, Batak, and Nias. Rice, coffee, and palm oil cultivation account for a large portion of the economy. Asahan also depends largely on its fishing industry.

The province of NTB lies just east of Bali with a population of about 4.7 million, of which 16 percent live below Indonesia’s village poverty line [20]. Lombok Utara is the northernmost district of NTB’s main island with a population of about 200,000. Lombok Tengah is directly below it and has a population of about 900,000. Both districts have a Muslim, ethnically Sasak majority. Rice farming is the principal source of income for most households, though many supplement their income by fishing, weaving, or working in the tourism industry. NTB is also home to many migrant workers, usually with jobs in domestic labor in countries such as Malaysia and Saudi Arabia.

Poor road networks, together with seasonal flooding and inadequate public transportation, restricted access to the district capitals for many community members in the four districts.

### Data collection and sample

Data collection was led by the Center on Child Protection and Wellbeing at Universitas Indonesia (PUSKAPA) and Columbia University. Focus group discussions were conducted in Bahasa Indonesia by a PUSKAPA researcher with a lead researcher and a note taker present. All sessions were also recorded digitally. After each session, the team reviewed the recordings and field notes and held a debriefing session to address issues during data collection.

Focus groups included an adapted participatory ranking methodology (PRM) exercise to engage participants more actively during data collection while generating rich and contextualized data [21]. The framing question was “what barriers to obtaining a legal identity document do communities identify as being the most critical to overcome.” In the PRM, participants ranked these barriers in order of priority. Focus groups in each district were stratified into four groups. The first consisted of district officials from the Office of Population and Civil Registration, the Office of Religious Affairs, and the Religious Court. Officials were included in the study if their primary job responsibility involved delivering legal identity services. The second group consisted of members from an all-female civil society organization (CSO) who provided information and support to community members applying for birth and marriage certificates, as well as marriage legalization for religiously married couples who had their children before being legally married. These CSO members also helped the research team to identify participants for the other two strata: male and female community members who were married with children.

### Ethical considerations

Atma Jaya University’s Ethical Review Board reviewed and approved the study protocols. After the purpose of the study was explained to them, all participants provided written, informed consent agreeing to participate in the study and have their voices recorded. Participants were not paid, but were thanked with small gift bags including school supplies or groceries worth USD 5, which was deemed appropriate for the context.

### Analysis

All of the recordings were transcribed verbatim. Research assistants who were present during data collection verified and corrected all transcripts. Half of the transcripts were translated into English to allow the Columbia researchers to guide the PUSKAPA researchers through the first phases of analysis. In-country researchers also received training on Nvivo10 [22]. The analysis team, which consisted of three researchers from Columbia and five researchers from PUSKAPA, reviewed the English transcripts first to resolve any issues with the translation and then to familiarize themselves with the data. The team then open-coded a sample of the English transcripts in Nvivo10. Using constant comparative method, the team reviewed their open-codes, held discussions, wrote memos, and developed an initial codebook [23]. The team then applied the initial codebook to the entirety of the English transcripts, refining the codes and definitions iteratively. After resolving discrepancies in the codes and the application of the codebook, the PUSKAPA team coded the remaining Indonesian transcripts. Without reference to a preexisting theory or model, the codes were then classified into different categories corresponding to larger themes in the data. In examining the categories and themes, the research team found significant overlap with the factors and phases of the Three Delays Model. The data were then revisited using thematic analysis to inform the results presented in this article.

## Results

A total of 20 focus group discussions (FGDs) were conducted in 11 villages across the four districts. The research team met with four groups of district-level government officials, including providers of birth and marriage certificates and judges who were responsible for presiding over marriage legalizations and divorces. Most of these officials were men. The two CSO focus groups consisted entirely of female paralegals with a median age of 43. Most participants in the seven FGDs with the female stratum of community members identified as housewives. Their median age was 36. Of the seven FGDs with male community members, most were farmers, with some working as fishermen, particularly in SUMUT. Their median age was 44. Despite generally low birth certificate ownership in these villages, the majority of the participants’ children had birth certificates.

Results from the FGDs indicated that financial issues, access to information, cultural beliefs and practices, logistics, and perceived poor quality of services posed major challenges for communities to register their children's births (Table 1). Of all the barriers to registration, participants across strata mentioned financial issues the most frequently, with the exception of male community members. The complex procedures and requirements for registration were also major concerns for community members and CSO members. Participants also mentioned the lack of information about registration procedures as a major barrier, especially in NTB.

**Table 1:** Most Common Barriers to Civil Registration in SUMUT and NTB, Based on PRM.

	Government Officials	CSO	Female Community Members	Male Community Members	Totals	
Categories	Freq	Freq	Freq	Freq	Freq	%
**Financial**	5	1	7	4	**17**	**85**
**Procedures and requirements**	0	2	5	3	**10**	**50**
**Lack of information**	1	1	3	4	**9**	**45**
**Quality of services**	1	1	2	2	**6**	**30**
**Time needed for application**	0	0	3	2	**5**	**25**
**Cultural practices**	1	0	1	3	**5**	**25**
**Distance**	1	0	0	1	**4**	**20**
**Perception and motivation**	3	0	0	1	**4**	**20**

**Fig 1:**
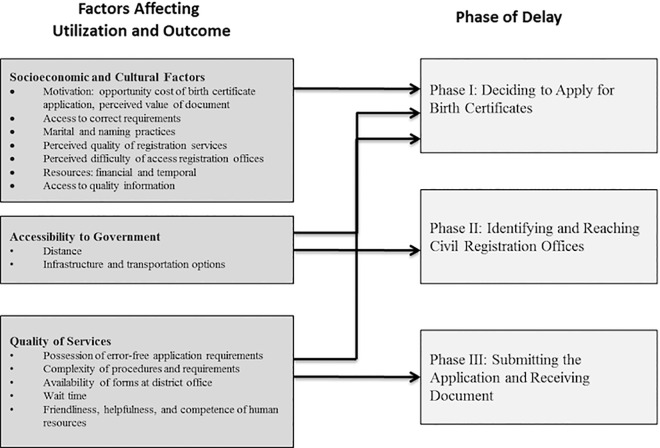
Adaptation of the Three Delays Model for Civil Registration.

The following sections describe how these categories contribute to themes and specific delays in birth registration (Fig 1).

### Phase I: Deciding to Apply for Birth Certificates

The first delay phase, the decision to apply for a birth certificate, reportedly occurred for a number of socioeconomic and cultural reasons, such as the availability of resources, local traditions, and the requirement of a birth certificate to access services, such as schooling.

Across strata, most participants believed that birth certificates were important for their children’s futures. They attributed this importance to the use of certificates for specific administrative activities, typically school enrollment and passport applications for the hajj pilgrimage or migratory work. Parents in all study areas, however, often did not begin applying for birth certificates until they were needed for one of these activities. As one female participant from SUMUT explained,

“*Children need to have a birth certificate in order to continue their education… They only start applying for the certificate when they are about to enroll in high school*.”

Knowing the tendency for community members to delay their marriage and birth registration applications, religious court officials in SUMUT even had a policy of expediting marriage legalization cases that could enable family members to go on hajj. As one official confirmed,

“*The ones applying for hajj pilgrimage are sometimes prioritized*.”

Parents had several reasons for delaying the application process. Many participants claimed they could not afford the costs associated with registration, such as transportation to registration offices, duty stamps for official letters, and photocopies of required documents. With insufficient income to pay for their family’s various needs, parents weighed the opportunity costs of these purchases against more immediate needs. As one participant narrated,

“*My daughter needed to get medical treatment*. *She had an ear infection and a tonsil problem*. *Which one is more important*? *Surely getting the medical treatment is more important than getting the certificate*.” (Female community member, SUMUT).

Community members also reported finding the registration process complex, confusing, and time-intensive. Many did not know how to start an application or what was required. As one CSO member from SUMUT described, “*Some parents have no idea how or where to acquire certificates*.” Participants often attributed the lack of knowledge about registration procedures to low educational attainment in their communities. According to one male participant from NTB,

“*Many village people don’t go to school*, *they don’t dare to* [apply for documents] *by themselves*, *and nor do we*. *Most of the people in the sub-villages are like that*.”

Others thought the problem resulted from insufficient efforts by civil registration officials and other authorities to inform communities about the official registration process. As one male participant from SUMUT opined,

“*They should explain the process well and make it easy*. *If they did that*, *then maybe all of us would have certificates*.”

Obtaining a marriage certificate–which until early 2016 was required in order to include the father’s name on a child’s birth certificate–was particularly challenging for many parents. This challenge was especially significant in NTB, where polygamy is common and remarrying several times is customary. Rather than paying the costs of divorce and remarriage, couples simply forego registering their marriage. One male participant from NTB explained,

“*I’ve gotten married five times*, *but no marriage certificate at all*. *Not even one*.”

Without the parents’ marriage certificate, children were provided birth certificates without a father’s name, which many community members found stigmatizing. Not having a marriage certificate delayed birth registration because many unregistered couples did not want their children’s birth certificates to exclude the father’s name. One CSO member recounted the following story about a second wife in a polygamous marriage who had asked for help registering her child:

“*She wanted to obtain a birth certificate for her child because her child wanted to go to school*. *When the birth certificate was issued*, *the child was enrolled in the school*, *but the teacher asked why the father’s name was not on the birth certificate*. *The wife came to me and asked me* [why that was the case] *and I said because she had no marriage certificate as required for a* [complete] *birth certificate*, *and told her to explain that to the teacher*. *The officer from registration office does not want to issue birth certificates with the mother and father’s name if the parents do not have a marriage certificate*. [The mother] *looked agitated*, *upset*, *and disappointed*.” (CSO member, SUMUT).

Many participants also mistakenly believed that marriage certificates were mandatory for registering their children’s births. As one male participant from SUMUT explained,

“[S]*ome people only have a religious wedding and thus do not have a marriage certificate*, *and cannot apply for* [their children’s] *birth certificates*.”

The legalization process that couples without marriage certificates had to undergo at the religious court in order to produce a birth certificate with both parents’ names required them to bring witnesses, visit the court several times, and, in many cases, spend additional money. As one female participant from SUMUT explained,

“*They tell us to bring a witness*. *That means we have to pay for the witness*. *I understand the need for a witness but I don’t like the idea of having to pay for the witness to go to court*. *I need that money*.”

Marriage certificates were not the only problematic requirement. Many parents had expired identity cards or had not procured the appropriate documentation, such as a birth notification letter. In SUMUT, for example, a migrant family originally from Lombok was turned away when applying for their family card, which is required for a child’s birth certificate. They had not known to update their residential status with an official migration letter before travelling to the registration office. As a CSO member recounted,

“*So this person migrated from Lombok and wanted to apply for a family card*. *But this person was asked to return to Lombok to obtain a statement letter for relocation*. *This person does not have any money; thus*, *the person does not own any family card up to this day*.” (CSO member, SUMUT).

These additional complications factored heavily into decisions to seek birth registration services.

### Phase II: Identifying and Reaching Civil Registration Offices

Once parents decided to register their children’s births, they had difficulty accessing registration offices in the district capital. The delay in accessing services at government offices was primarily due to a combination of factors regarding information, infrastructure, and resources.

Residents in rural areas rarely had contact with the district capital. Many did not know which offices to visit, let alone where they were located. As one male participant from NTB noted,

“*We lived in the sub-village and* [birth certificates] *are made in the city*. *We don’t even know where the office is*.”

Participants in all study areas reported that the distance to the registration offices and the time needed for the commute delayed birth registration. One community in NTB even held demonstrations to request services closer to their village. Applicants in Langkat had to drive up to three hours each way to reach the registration offices. As one male participant from SUMUT elaborated,

“*The problem is that the distance is too great*. *We don’t have the money*, *and we are too busy to make the time*.”

Poor roads and a lack of public transportation made trips to civil registration offices an uncomfortable and costly endeavor. As one CSO member from NTB reported,

“*Some roads there are still in a poor condition*, *and also*, [applicants] *have to go back and forth by motorbike taxi*. *People who don’t have motorbikes take days to process* [their certificates].”

To avoid commuting to district offices, communities submitted their applications to one individual, usually the village leader, who then delivered the applications to the registration office. These individuals usually charged a transportation fee, but, in order to reduce costs, tended not to submit the applications until they had several at once. This created a further registration delay. As one male participant from SUMUT reported,

“*I had to pay money* for [the village leader’s] *transportation and accommodations*. *The official here had to wait until he had enough applicants before he could go to* [the district capital].”

In addition to belaboring birth registration, these obstacles also fed back into the first phase delay, as they discouraged people from wanting to apply in the first place.

### Phase III: Submitting the Application and Receiving Document

Those who managed to overcome the challenges above often faced additional challenges after arriving at district registration offices. Errors in required documents were common, such as a misspelled name or an incorrect date. Because of strict policy regulations and input procedures within the civil registration management information system, civil registration providers rejected applications with spelling or date inconsistencies between documents. As one participant recalled,

“*I once saw this at a civil registration office*, *far away*. *This person was there from morning to evening and yet her certificate wasn’t finished*. *She cried and begged*. *She only had a little mistake and they refused to help her*.” (Female community member, SUMUT).

According to participants, many of these inconsistencies resulted from simple clerical errors in the documents required for registration applications, while others were influenced by cultural practices. In Lombok, parents traditionally add their first child’s name to their own, and also change their names when they make the pilgrimage to Mecca. If parents use their new names on any of the documents required for the newborn’s birth certificate, the application can be rejected. Furthermore, if this happens and the officer does not catch the discrepancy, birth certificates may be printed with the parents’ non-legal names, potentially creating problems in the future.

Acceptance of child marriage by local leaders was another cause of inconsistent documents. One CSO participant described an underage couple that had wedded without registering their marriage. When they wanted to produce a birth certificate for their child, they falsified their age to comply with Indonesia’s legal age of marriage. As one CSO member from NTB explained, if child applicants are already religiously married, sometimes officer

“*mark up their age—even if the kids are still underage—to get* [officially] *married*.”

Many community members reported that they could not submit their applications even when they had all the requirements. Offices were often understaffed and did not have enough blank certificates available, and several reportedly closed early. One CSO member from NTB shared a typical experience:

“*Even to submit the requirements*, *we have to queue for the whole day*, *and we would still not necessarily get our turn on that day*. *Sometimes we have to wait until the day after*, *and go back and forth again*.”

Participants reported that, once they submitted their applications, they waited weeks and sometimes months before picking them up. Registration officials reportedly had inadequate human resources and budget available for processing the volume of applications they received. Officials felt the practice of waiting until a birth certificate was required to begin the application made community members rush unnecessarily through the process. As one official from SUMUT said,

“[t]*here are always people demanding immediate services every day*. *If possible*,*”*

the official continued, applicants want their birth certificates

*“processed in a day*. *It would be even better for them if we were able to process* [their applications] *within half an hour*.”

Community members reported that there was no system to notify applicants when documents were ready. Applicants had to check their status with the officer repeatedly, often in person. As one female community member from SUMUT said,

“*I don’t mind waiting if there is a certainty that the birth certificate will be issued*. *Whether it’s three days or one week*. *What I don’t like is having to go to the office all the time* [to check on the status of my application].”

Bribes, solicited and unsolicited, were reported as being used to expedite services across study sites. One male participant from NTB expressed a common sentiment that community members were

“*afraid their documents would not be processed if they don’t give a tip*.”

Participants in SUMUT reported the same tendency. As one male community member explained,

“*You need to pay the money first*. *All the other requirements such as copies of marriage certificates*, *family cards*, *and such things can follow later*, *so long as you pay them some money*.”

Poorly communicated information, errors in printed documents, long wait times, and a lack of transparency in the application process were interpreted by many as poor quality service. This inadequate service also compounded the Phase II delay, as applicants were forced to travel to registration offices several times to complete their applications or to check their application status. The expectation of poor quality service and the costly and lengthy travel times in turn fed back into Phase I delays, in which parents decided to forestall registering their children’s births.

## Discussion

### Applicability of the Three Delays Model to Civil Registration

These results demonstrate the relevance of Thaddeus and Maine’s model to civil registration in rural SUMUT and NTB. The focus group discussions confirmed findings from previous studies of civil registration in Indonesia, where cost, distance, application information, and a complex application process obstructed access to birth certificates for children in poor and rural settings [5]. Unsurprisingly, financial barriers were the most frequently mentioned and often the highest ranked factor in the PRM exercise. By examining these barriers as part of an integrated framework, the study also found that the significance of the different barriers varied not only according to the applicant’s socioeconomic standing, but across the child’s life course as well.

Without question, the cost of birth certificates discouraged many parents in low-income households from registering births during the first few years of a child’s life. Because Indonesian authorities and service providers rarely required young children to have birth certificates to benefit from government programs, parents often preferred to spend their money on more immediate concerns, such as medical care. In fact, many participants said that they delayed registering their children’s births until the certificate became valuable to them, such as during school enrollment, even if it meant paying more money.

Parents’ willingness to spend more money for delaying birth registration may help to explain why previous attempts to remove application fees and impose late-registration fines did not lead to rapid increases in birth registration [4, 20]. As children grow older, their birth certificates confer more benefits, and so the monetary investment becomes more worthwhile for their parents. A UNICEF review of data from 161 countries found that birth certificate coverage tended to increase with age around the world, in part because schools and health providers required them [1]. A recent study of three sub-districts in Indonesia found a similar trend, though it also noted that there was no sizeable increase in the proportion of children receiving a birth certificate between the ages of four and six, as would be expected if initial school enrollment were the principal motivating factor for registration [24]. While birth certificates are not required to access healthcare or social protection benefits in Indonesia, their use for creating school diplomas, and for scholarship applications, marriage certificates, and passports contribute to their increasing value as children age.

This finding suggests that in order to address Phase I delays and achieve improvements in the demand for timely birth registration, authorities should focus on enhancing the value of birth certificates earlier in children’s lives rather than on deterrence policies that increase the financial burden of applying, such as late registration fees. Connecting birth certificates more closely to programs that directly benefit young children would ensure that registration becomes valuable to parents earlier in their children’s lives. At the same time, requiring that children present birth certificates to benefit from basic services risks excluding the most marginalized populations from those services, especially considering the challenges that many families face in accessing civil registration services [3, 24]. In an effort to incentivize early registration without adversely affecting children’s access to basic services, some initiatives in Indonesia have created special benefits for children with birth certificates, such as discounts on goods related to school, health, and extracurricular activities, though it is still unclear whether these benefits have contributed to increased demand for registration [25].

Applying the Three Delays Model also helps to parse the multiple effects that single factors may have on birth registration. The long distance to services for instance directly created delays in registration. If an applicant had to drive three hours to the district registration office to submit an application, rather than submitting it locally, that was three hours that the registration official was delayed in reviewing the application. The expectation of long drives also led parents to delay preparing or submitting their applications. By implication, an investment in reducing the distance between applicants and services should have a dual benefit: reducing the delay in applying for birth certificates and in processing them. The finding that parents’ distance from registration services had multifold effects on their registration delays provides support for the model of delivering civil registration to the sub-district and village levels through integrated and mobile services. It also supports policies that provide authority for registration to officials below the district level [4, 24].

Similarly, the framework of delays helps to reveal feedback effects between barriers. In Phase I, the decision to delay birth registration until the moment a child needed the certificate led parents to rush the application process. This in turn led to complications with Phase III, including errors in application forms, dissatisfaction with processing times, and negative exchanges with registration officials. These experiences then discouraged future applications, contributing to the Phase I delays. Examining the relationship between these barriers provides a deeper understanding of how communities experienced the registration process, with important insights for civil registration strengthening programs. One implication is that efforts to build demand for birth registration, for instance by increasing community awareness about the uses of birth certificates, are likely to be hamstrung without commensurate efforts to improve the supply of capable and friendly registration staff.

### Applying Approaches in Primary Health Programs to Improve Civil Registration

Applying the Three Delays Model to birth registration encourages cross-sector learning. While this article does not intend to compare obstetric care with civil registration, the several decades of experience tackling the barriers of distance, cost, and quality of care in the reproductive health field can be adapted to inform effective interventions for civil registration. Using a shared framework highlights the natural overlap between birth delivery and birth registration and promotes leveraging existing health approaches, such as voucher systems to overcome transportation barriers for the poor [26].

Crossover already occurs between primary health and civil registration. In Ghana and Ethiopia, community health workers were trained to register births to reduce the transportation burden in remote areas [27–29]. In Bangladesh, birth registration was incorporated into the Expanded Program for Immunization, and health workers helped to collect and transfer birth certificate application materials [27]. UNICEF has worked with dozens of countries in similar ways to integrate birth registration into primary health facilities and the provision of routine health services [27].

Primary health interventions are also relevant to the Indonesian civil registration system.

The country’s civil registration system parallels and often intersects with the primary healthcare system, creating opportunities for the two systems to mutually reinforce one another. As a result of efforts to reduce rural, maternal mortality dating back to 1989, midwives are often the most accessible healthcare provider for households in Indonesia [24, 30]. In addition to attending and reporting births and deaths within health facilities as well as in private homes, midwives are also responsible for signing birth notification letters, which are required for birth certificate applications. Despite their convenient access to birth certificate applicants, health staff are not authorized to register births and do not have any official responsibility for supporting birth registration [24]. Still, mothers sometimes ask their attending midwives for counsel or assistance with birth registration procedures as there are typically no civil registration service providers available in villages or even sub-districts. One study found that in-facility births, births delivered by a skilled health attendant and accessing postnatal care in Indonesia were all associated with child birth certificate ownership [10].

In many districts and cities across Indonesia, local governments have started to formalize the relationship between the health and civil registration sectors, whether through local regulations or formal cooperation agreements. In the city of Surakarta, staff at hospitals and birthing centers facilitate birth registration and input birth data into the population registry [25]. In the district of Bireuen in Aceh, health workers help patients prepare certificate applications, and completed birth certificates are distributed through health facilities [31]. The Australian Department of Foreign Affairs and Trade’s Governance for Growth (KOMPAK) program is collaborating with the Ministry of National Development Planning (BAPPENAS) and the Center on Child Protection and Wellbeing at the University of Indonesia to learn from these examples and develop reproducible models for institutionalizing civil registration and vital statistics within basic services systems, including the healthcare system [24]. For the moment, however, the Ministry of Health in Indonesia remains completely independent of the Ministry of Home Affairs’ civil registration activities. Further fostering relationships between these ministries could be particularly useful for the Indonesian context and should be explored further.

The applicability of the Three Delays Model to connect primary healthcare and civil registration does have limitations. While the Three Delays Model in this study looks at birth certificate ownership, registration is generally an intermediary output to access other social services like education. Thus, improving the accessibility and the quality of the civil registration system does not automatically translate to increasing the utilization and positive outcomes of health or other basic services. This diverges from the healthcare context, where improving the quality of obstetric services directly affects the ultimate outcome of reducing maternal mortality. As such, reducing delays such as Phase I’s “decision to seek care or services” may be more difficult to achieve in the case of birth certificate ownership, whose negative outcome is not as severe as delaying obstetric care. The different implications of the results found by using the Three Delays Model should be accounted for when determining solutions to overcome barriers and similarly to adjust expected outcomes for reducing maternal mortality versus increasing birth certificate ownership.

### Limitations

Limitations for this study are primarily due to our selection of study sites and participants. The CSO members who helped the research team identify study participants also assisted community members to apply for birth certificates and played a role in the delivery of these services. This selection method likely left out more vulnerable community members, who were unknown to the CSO workers, and whose participation may have identified additional barriers.

The majority of the study participants had registered their children’s births. Participants were thus fairly aware of the civil registration system and how to navigate it successfully. Part of this knowledge may have been due to the site selection. All of the communities visited had received civil registration awareness campaigns and integrated mobile service events to register children. Although none of the participants had directly accessed the mobile service units, there may have been a spillover effect making them more aware of the importance of and requirements for birth certificates. Our sample may have had greater knowledge of the legal identity process and perceived importance of documents. Attempts were made to reduce this bias by also including randomly selected community members who participated in a household survey simultaneous to this research.

Finally, this study cannot be generalized to the broader Indonesian civil registration system as it excluded important aspects of that system, most notably death registration. The Three Delays Model may be useful in characterizing the challenges related to death registration, especially considering the health sector’s traditional responsibility of ascertaining and certifying cause of death. Such an analysis is beyond the scope of this article.

## Conclusions

Identifying and understanding the relationship between the barriers to accessing high quality social services is crucial. The Three Delays Model, originally developed to conceptualize factors contributing to maternal mortality, helps capture this complexity in the Indonesian civil registration system. Dividing the process of applying for civil registration documents into three phases offers useful insight for policies to promote civil registration ownership. Ultimately, in order to reach its ambitious target of registering 85 percent of children’s births by 2019, Indonesia’s government will likely need to formalize the role of the primary healthcare system in working with the civil registration sector [6, 24]. Not only do their shared factors affecting utilization and outcome of public services help inform programs, but the facilities and staff supporting maternal, neonatal, and child care also present an indispensable opportunity to incorporate birth registration as a basic service. As Indonesia renews its commitment to civil registration and population health under its National Medium Term Development Plan and the SDGs, this inter-sectorial work may consolidate efforts to achieve both of these objectives.
